# Supplementary material for: CD276-dependent efferocytosis by tumor-associated macrophages promotes immune evasion in bladder cancer
Source: Nat Commun. 2024 Apr 1;15:2818. doi: 10.1038/s41467-024-46735-5 (PMC10985117; doi:10.1038/s41467-024-46735-5)
Supplement: Supplementary file 1 — Supplementary Information [file 41467_2024_46735_MOESM1_ESM.pdf]

CD276-dependent efferocytosis by tumor-associated macrophages promotes immune evasion in bladder cancer

Maosheng Cheng[1*], Shuang Chen[1*], Kang Li[1*], Ganping Wang[2*], Gan Xiong[1*], Rongsong Ling[3], Caihua Zhang[1], Zhihui Zhang[1], Hui Han[1], Zhi Chen[1], Xiaochen Wang[1], Yu Liang[1], Guoli Tian[4], Ruoxing Zhou[1], Yan Zhu[1], Jieyi Ma[1], Jiahong Liu[5], Shuibin Lin[1#], Hao Xu[6#], Demeng Chen[1#], Yang Li[7#], Liang Peng[5#]

1. Department of Medical Oncology; Institute of Precision Medicine; Center for Translational Medicine, The First Affiliated Hospital of Sun Yat-sen University, Guangzhou 510080, China.
2. Department of Urology, Zhujiang Hospital, Southern Medical University, Guangzhou, China.
3. Institute for Advanced Study, Shenzhen University, Shenzhen, 518057, China.
4. Hospital of Stomatology, Sun Yat-sen University, Guangzhou, China.
5. Senior Department of Oncology, the Fifth Medical Center of PLA General Hospital, NO.8 the east street, Fengtai District, Beijing, 100071, China
6. State Key Laboratory of Oral Diseases, National Clinical Research Center for Oral Diseases, Research Unit of Oral Carcinogenesis and Management, Chinese Academy of Medical Sciences, West China Hospital of Stomatology, Sichuan University, Chengdu, Sichuan 610041, China.
7. Department of Genetics, School of Life Sciences, Anhui Medical University, Hefei, 230031, China

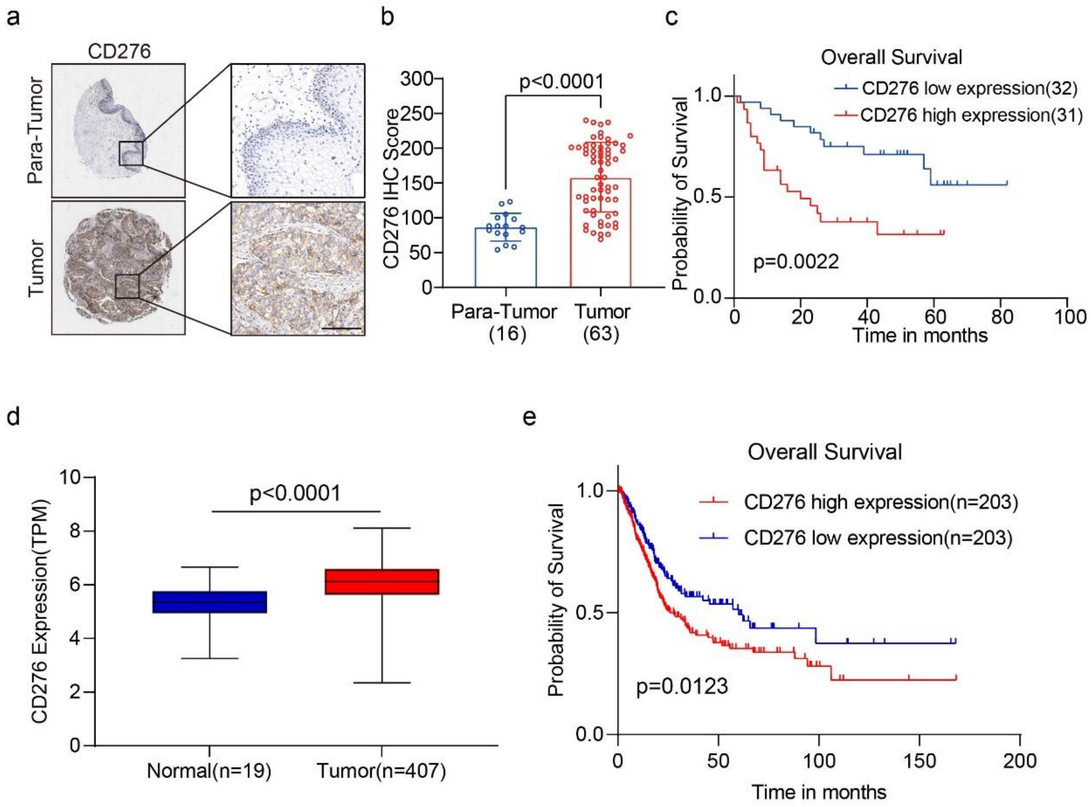

Supplementary Fig 1, Related to figure 1. CD276 is highly expressed in bladder cancer and leads to poor prognosis.

a, b Representative images of CD276 IHC staining (a) and quantification of CD276 IHC score (b) in tumor (n = 63) and corresponding normal tissues (n = 16) in bladder cancer tissue microarrays. Scale bar,100μm. Data are presented as mean ± SD. *P* values were calculated by two-tailed unpaired Student's t-test.

c Kaplan-Meier overall survival from 63 bladder cancer patients according to CD276 expression level. *P* values were calculated by log-rank test.

d The mRNA level of CD276 in bladder urothelial carcinoma (BLCA) based on The Cancer Genome Atlas (TCGA) dataset. *P* values were calculated by two-tailed unpaired Student's t-test.

e Kaplan-Meier overall survival curves of individuals with different CD276 mRNA expression in the TCGA-BLCA dataset. *P* values were calculated by log-rank test.

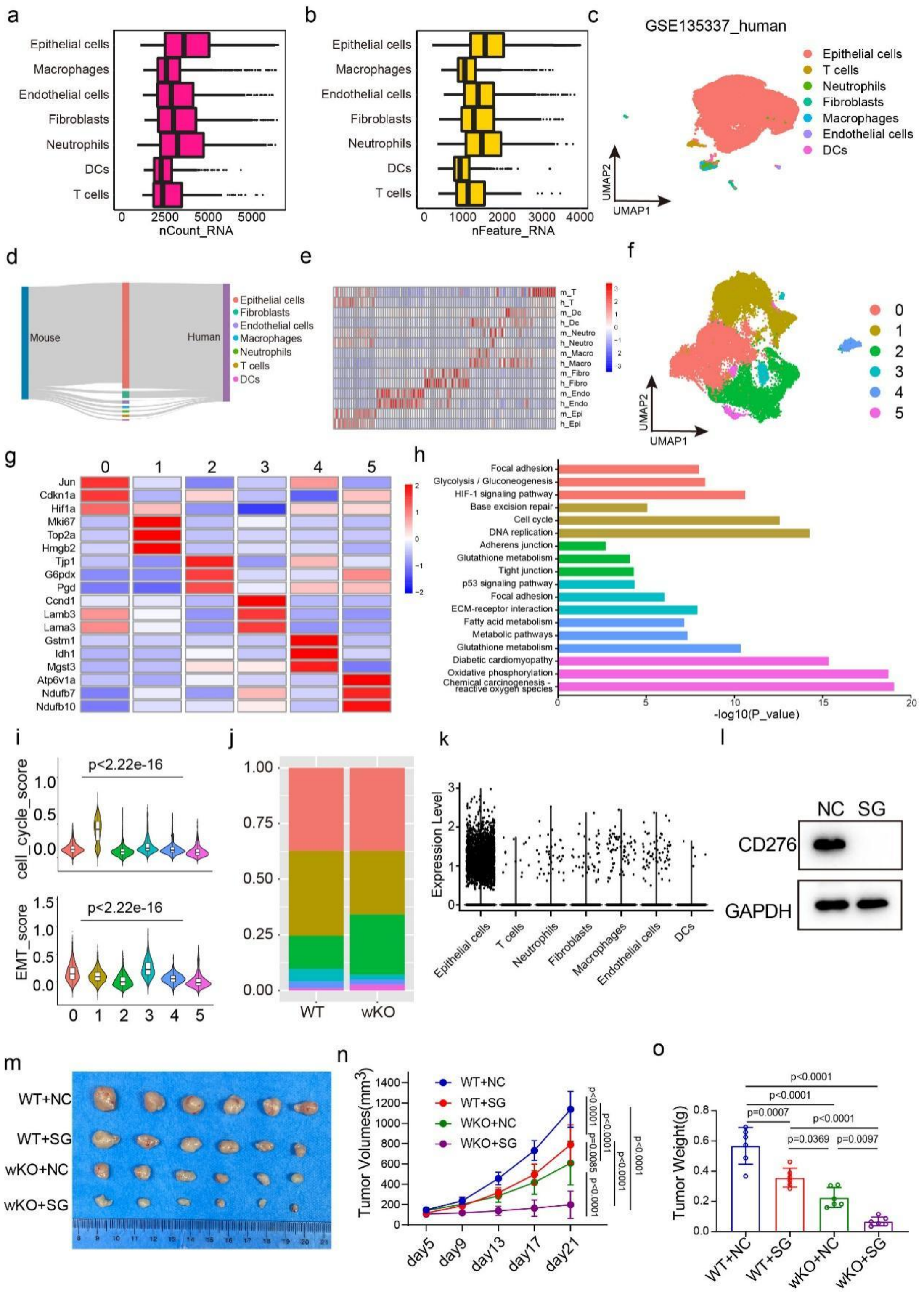

Supplementary Fig 2, Related to figure 2. Cd276 plays a more important role in stromal cells than tumor cells.

a, b Box plots of the number of UMIs (A) and genes(B).

c UMAP of single-cell clusters from human BLCA tumor tissues (n = 7, GSE135337), colored by cell cluster.

d Comparison of cellular profiles between human and mouse BLCA data (Sankey diagram). The

height of each linkage line reflects the proportion of cells.

e Heatmap showing genes similarly enriched within mouse and human cells clusters from male mice BLCA tumor tissues and human BLCA tumor tissues.

f UMAP plots showing the subclusters of epithelial cells.

g Heatmap of signature genes for epithelia cells clusters from mouse BLCA tissues. Each cell cluster is represented by three specifically expressed genes.

h KEGG pathway enrichment analysis using the characteristic genes of epithelial cells subpopulation.

i Violin plots showing the scores of EMT and cell cycle signatures for epithelial cells.

j Bar plots of proportion of the subclusters of epithelial cells.

k Violin plot depicts expression of CD276 in all cell type from mouse BLCA tumor tissues.

l The knockout efficiency of Cd276 were validated in MB49 cell line by WB analysis.

m Overview of tumors in xenograft male mice model subcutaneously implanted with CD276 wKO and WT MB49 BLCA cells.

n, o Tumor growth curves (n) and tumor weight (o) at the sacrifice of subcutaneous xenograft model. Data was shown as mean ± SD, n= 6. *P* values are presented by one-way ANOVA with Tukey's multiple comparison test.

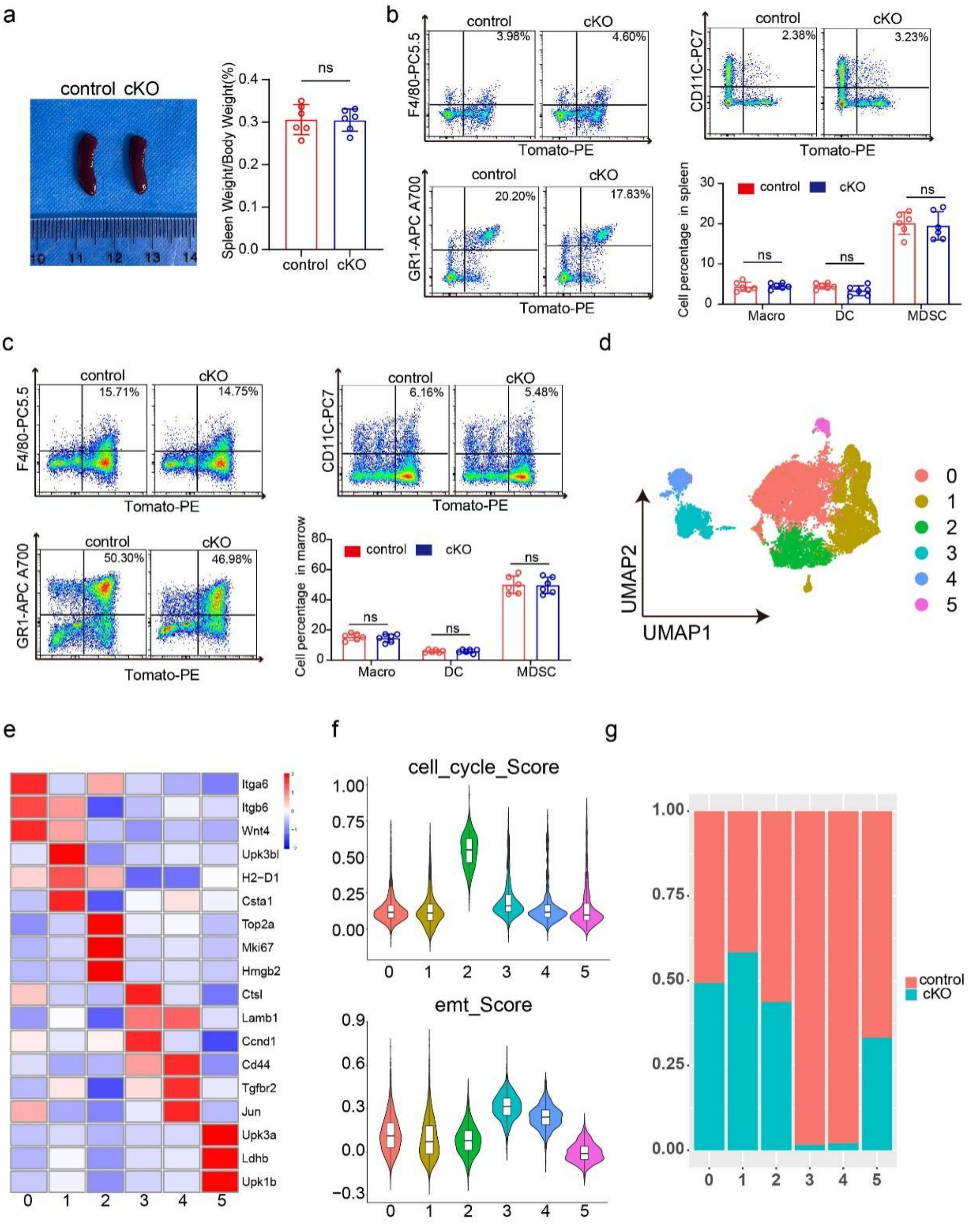

Supplementary Fig3, Related to figure 3. Loss of Cd276 in TAMs is tolerated under homeostasis but inhibits tumor progression.

a Representative images of normal spleen (left) and quantification of ratios of spleen weight to body weight (right) in control and cKO male mice. Data are presented as mean ± SD (n=6). *P* values were calculated by two-tailed unpaired Student's t-test. ns, no significance.

b Representative flow cytometry plots of normal spleen and population frequencies of macrophages, DCs and MDSCs in control and cKO male mice. Data was presented as mean ± SD (n=6). P values were calculated by two-tailed unpaired Student's t-test. ns, no significance.
c Representative flow cytometry plots of normal marrow and population frequencies of macrophages, DCs and MDSCs in control and cKO male mice. Data was presented as mean ± SD (n=6). *P* value was calculated by two-tailed unpaired Student's t-test. ns, no significance.
d UMAP plots showing the subclusters of epithelial cells.
e Heatmap of signature genes for epithelia cells clusters from mouse BLCA tissues. Each cell cluster is represented by three specifically expressed genes.
f Violin plots showing the scores of cell cycle (upper) and EMT (lower) signatures for epithelial cells.
g Bar plots of proportion of the subclusters of epithelial cells.

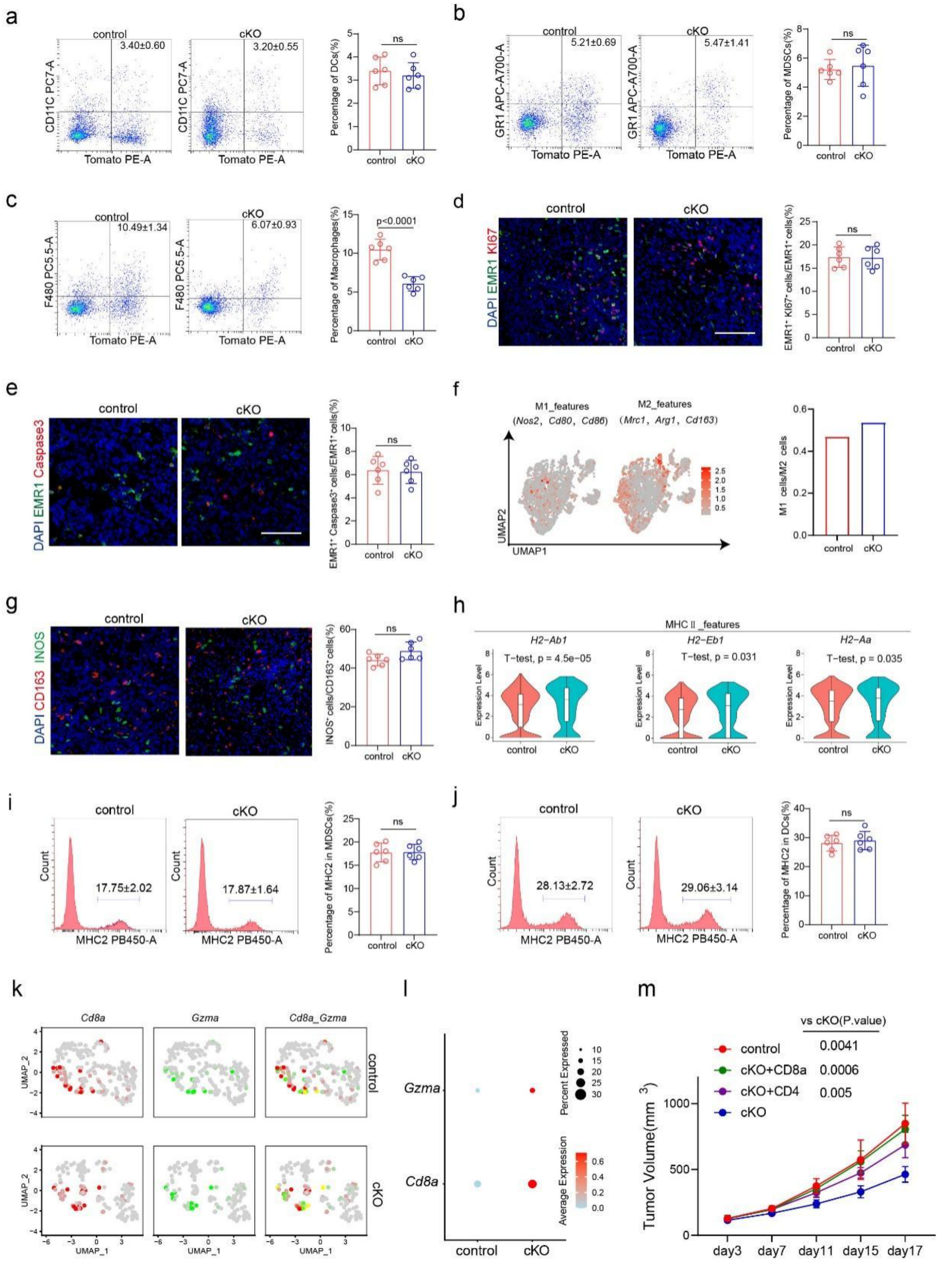

Supplementary Fig4, Related to figure 4. Loss of Cd276 in TAMs promotes antigen presentation and infiltration of effector CD8T cells.

a-c Representative flow cytometry plots and statistical analysis of DCs (a), MDSCs (b) and macrophages (c) in control and cKO groups. Data are presented as mean ± SD (n=6). *P* value was calculated by two-tailed unpaired Student's t-test. ns, no significance.

d Representative Immunofluorescence (IF) staining images of EMR1 (red) and KI67 (green)(left).

Statistical analysis of the radios of EMR1+ KI67+ cells to EMR1+ cells in control and cKO mice (right). Scale bar, 50μm. Data was presented as mean ± SD (n=6). *P* values was calculated by two-tailed unpaired Student's t-test. ns, no significance.

e Representative Immunofluorescence (IF) staining images of EMR1 (red) and Caspase3 (green)(left). Statistical analysis of the radios of EMR1+ Caspase3+ cells to EMR1+ cells in control and cKO mice (right). Scale bar, 50μm. Data are presented as mean ± SD (n=6). *P* values was calculated by two-tailed unpaired Student's t-test. ns, no significance.

f UMAP plots showing the expression of M1 feature gene sets (Nos2, Cd80, Cd86) and M2 feature gene sets (Mrc2, Arg1, Cd163). Quantification of ratios of M1 cells to M2 cells in control and cKO macrophages.

g Representative Immunofluorescence (IF) staining images of CD163 (red) and INOS (green) and quantification of ratios of INOS+ cells to CD163+ cells (right) in control and cKO mice. Scale bar, 50μm. Data was presented as mean ± SD (n=6). *P* values was calculated by two-tailed unpaired Student's t-test. ns, no significance.

h Violin plots showing the expression of MHCII signature genes (H2-Ab1, H2-Eb1, H2-Aa) in macrophages of control and cKO male mice.

i, j Representative flow cytometry plots and statistical analysis of MHCII+ cells in MDSCs (i) and DCs (j) from control and cKO groups. Data are presented as mean ± SD (n=6). *P* value was calculated by two-tailed unpaired Student's t-test. ns, no significance.

k UMAP plots of T cells color coded according to the expression of Cd8a and Gzma in control and cKO male mice.

l Dot plots showing the expression of Cd8a and Gzma in T cells of control and cKO male mice.

m Tumor growth curves at the sacrifice of subcutaneous xenograft model. Data was shown as mean ± SD, n= 6. *P* values were presented by one-way ANOVA with Tukey's multiple comparison test.

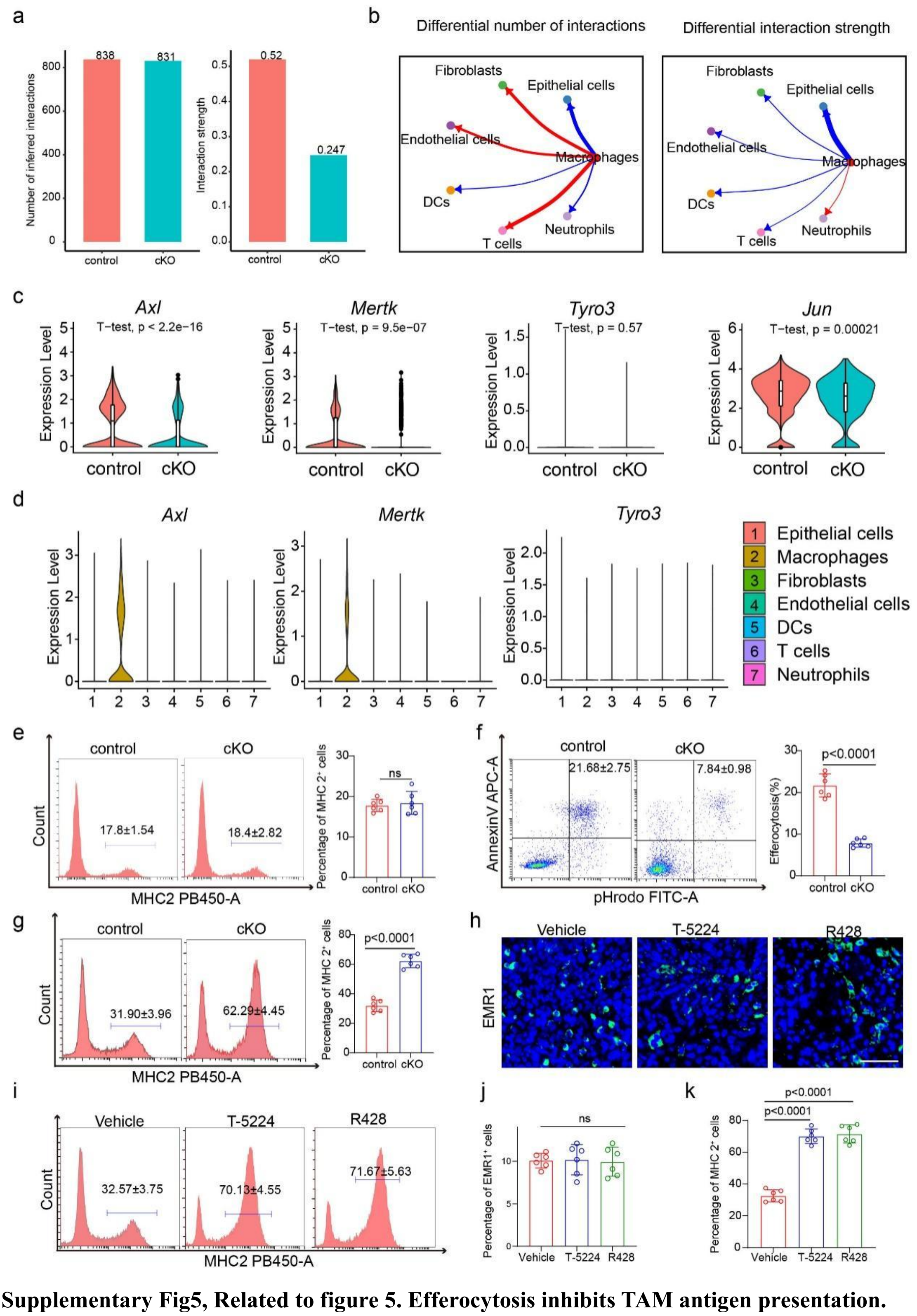

Supplementary Fig5, Related to figure 5. Efferocytosis inhibits TAM antigen presentation.

a Cellchat showing the overall number and strength of intercellular communication.

b Cellchat showing the difference of number and strength of intercellular communication between two groups. Both the strength and number of intercellular communication between macrophages and epithelial cells were significantly reduced.

c Violin plots showing the expression of Axl, Mertk, Tyro3 and Jun in macrophages of control and

cKO mice.

d Violin plots showing the expression of Axl, Mertk and Tyro3 in all cell types of male mice BLCA tissue.

e Representative flow cytometry plots (left) and statistical analysis of the percentage of MHCII+ cells (antigen presenting ability of macrophages, right) in control and cKO macorphages of marrow. Data was presented as mean ± SD (n=6). *P* value was calculated by two-tailed unpaired Student's t-test. ns, no significance.

f Representative flow cytometry plots (left) and quantification of percentages of efferocytosis in control and cKO macrophages of marrow (right). Data was presented as mean ± SD (n=6). *P* value was calculated by two-tailed unpaired Student's t-test.

g Representative flow cytometry plots (left) and statistical analysis of the percentage of MHCII+ cells (antigen presenting ability of macrophages, right) in control and cKO macorphages of marrow after coculture with apoptotic cells. Data was presented as mean ± SD (n=6). *P* value was calculated by two-tailed unpaired Student's t-test.

h, j Representative Immunofluorescence (IF) staining images of EMR1 of tumor (h) and quantification of percentages of EMR1+ cells in different treatment groups (j). Scale bar, 50μm. Data was shown as mean ± SD (n= 6). *P* values were presented by one-way ANOVA with Tukey's multiple comparison test. ns, no significance.

i, k Representative flow cytometry plots (i) and quantification of percentages of MHCII+ cells in macrophages of tumor (k). Data was shown as mean ± SD (n=6). *P* values were presented by one-way ANOVA with Tukey's multiple comparison test.

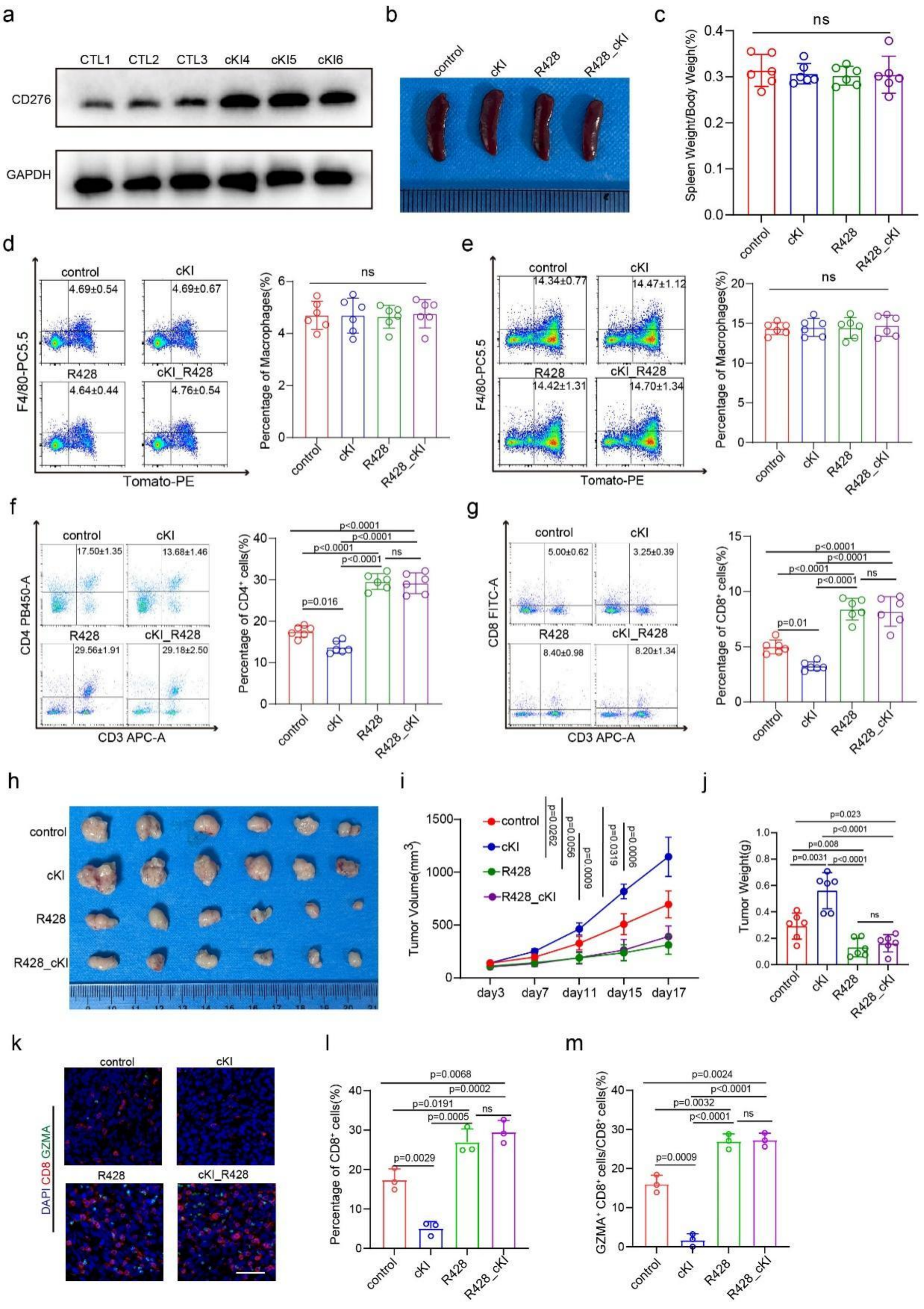

Supplementary Fig6, Related to figure 6. R428 is tolerated under homeostasis but inhibits tumor progression.

a Western blotting analysis of CD276 and GAPDH in BLCA macrophages of control (CTL) and cKI mice.

b, c Representative images of normal spleen (b) and quantification of ratios of spleen weight to body weight (c) in different treatment groups. Data were presented as mean ± SD (n=6). *P* values

were presented by one-way ANOVA with Tukey's multiple comparison test. ns, no significance.

d, e Representative flow cytometry plots of normal spleen (d) and marrow (e) (left) and population frequencies of EMR1+ macrophages (right) in different treatment groups. Data are presented as mean ± SD (n=6). *P* values were presented by one-way ANOVA with Tukey's multiple comparison test. ns, no significance.

f, g Representative flow cytometry plots and statistical analysis of CD3+CD4+ T cells (f) and CD3+CD8+ cells (g) in different groups. Data are presented as mean ± SD (n=6). *P* values were presented by one-way ANOVA with Tukey's multiple comparison test. ns, no significance.

h Overview of tumors in xenograft mice model subcutaneously implanted with MB49 BLCA cells in different treatment groups.

i, j Tumor growth curves (i) and tumor weight (j) at the sacrifice of subcutaneous xenograft model. Data was shown as mean ± SD, n= 6. *P* values were presented by one-way ANOVA with Tukey's multiple comparison test. ns, no significance.

k, l Representative Immunofluorescence (IF) staining images of CD8 (red) and GZMA (green, k) and quantification of percentages of CD8+ cells in xenograft (l) of different treatment groups. Scale bar, 50μm. Data was shown as mean ± SD(n=6). *P* values were presented by one-way ANOVA with Tukey's multiple comparison test. ns, no significance.

m Statistical analysis of the radios of CD8+ GZMA+ cells to CD8+ cells in xenograft of different treatment groups. Data was shown as mean ± SD (n=6). *P* values were presented by one-way ANOVA with Tukey's multiple comparison test. ns, no significance.

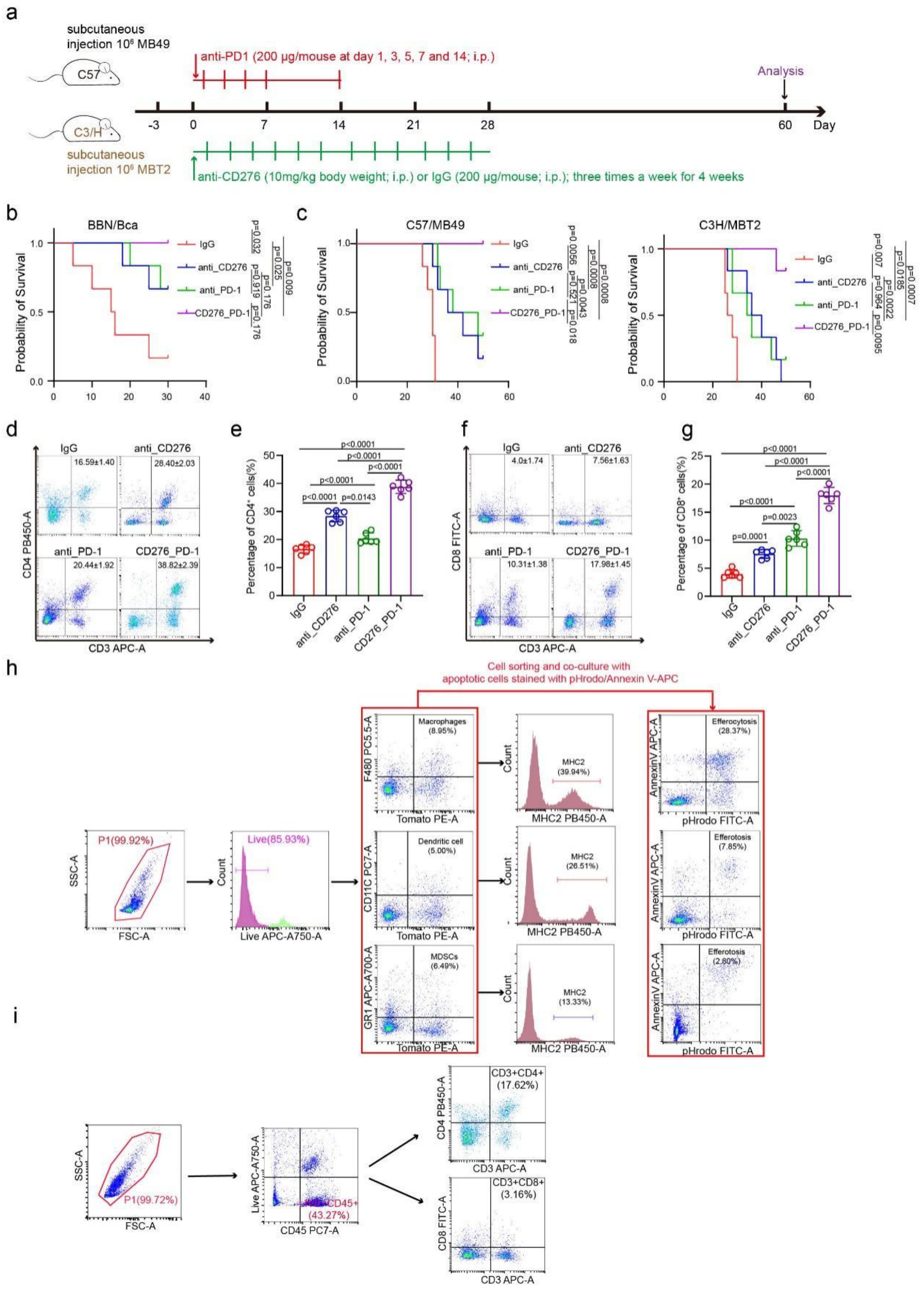

Supplementary Fig7, Related to figure 7. CD276 combined with PD-1 antibody therapy can improve prognosis of BLCA mice.

a Subcutaneous MBT2/C3H and MB49/C57 bladder cancer model. At day 3 after injection, the male mice with similar tumor volumes were randomly divided into four groups (six mice per group) for i.p. treatment with IgG (200 μg/mouse at day 1, 3, 5, 7 and 14), anti-PD1 (200 μg/mouse at day 1, 3, 5, 7 and 14) , anti-CD276 (10mg/kg body weight; three times a week for 4

weeks) or anti-PD1 plus anti-CD276.

b Kaplan-Meier curve of the overall survival rate in different treatment groups of BBN-induced BLCA. *P* values were calculated using the log-rank test.

c Kaplan-Meier curve of overall survival in different treatment groups of C57 tumour-bearing male mice injected with MB49 cells (left) and C3H tumor-bearing male mice injected with MBT2 cells (right). *P* values were calculated by log-rank test.

d-g Representative flow cytometry plots (d, f) and statistical analysis of CD3+CD4+ T cells (e) and CD3+CD8+ cells (g) in different groups. Data are presented as mean ± SD (n=6). *P* values were presented by one-way ANOVA with Tukey's multiple comparison test.

h Sequential gating strategy of myeloid cells from tumor tissue for flow cytometry analysis. The respective gate names are given in the corresponding figures.

i Sequential gating strategy of lymphocytes from tumor tissue for flow cytometry analysis.
