# Supplementary material for: CD276-dependent efferocytosis by tumor-associated macrophages promotes immune evasion in bladder cancer
Source: Nat Commun. 2024 Apr 1;15:2818. doi: 10.1038/s41467-024-46735-5 (PMC10985117; doi:10.1038/s41467-024-46735-5)
Supplement: Supplementary file 3 — Description of Additional Supplementary Files [file 41467_2024_46735_MOESM3_ESM.pdf]

Description of Additional Supplementary Files

Supplementary Data 1：93 tumor tissue specimens from BLCA patients who underwent surgical treatment at the Fourth Medical Center of PLA General Hospital.

Supplementary Data 2 a commercial tissue microarray containing 63 BLCA and 16 para-tumor tissues from Shanghai OUTDO BIOTECH Co., Ltd.

Supplementary Data 3：Differential gene analysis revealed 39 up-regulated genes and 99 down-regulated genes in CD276-wko epithelial cells.

Supplementary Data 4：Differential expression genes (DEGs) in TAMs after CD276 ablation were identified by pseudobulk analysis. 331 up-regulated genes and 492 down-regulated genes were identified in CD276 cKO-TAMs compared to the control group.

Supplementary Data 5：Evaluation of the AUC score of each potential factor by SCENIC analysis for determination of the transcriptional activity of transcription factors in TAMs.

Supplementary Data 6：list of primers used for this manuscript.
