# Supplementary material for: CD276-dependent efferocytosis by tumor-associated macrophages promotes immune evasion in bladder cancer
Source: Nat Commun. 2024 Apr 1;15:2818. doi: 10.1038/s41467-024-46735-5 (PMC10985117; doi:10.1038/s41467-024-46735-5)
Supplement: Supplementary file 10 — Reporting Summary [file 41467_2024_46735_MOESM10_ESM.pdf]

nature portfolio

Corresponding author(s): Shuibin Lin, Hao Xu6, Demeng Chen, Yang Li, Liang Peng

Last updated by author(s): Sep 30, 2023

Reporting Summary

Nature Portfolio wishes to improve the reproducibility of the work that we publish. This form provides structure for consistency and transparency in reporting. For further information on Nature Portfolio policies, see our Editorial Policies and the Editorial Policy Checklist.

Statistics

For all statistical analyses, confirm that the following items are present in the figure legend, table legend, main text, or Methods section.

n/a	Confirmed	
☐	☒	The exact sample size (*n*) for each experimental group/condition, given as a discrete number and unit of measurement
☐	☒	A statement on whether measurements were taken from distinct samples or whether the same sample was measured repeatedly
☐	☒	The statistical test(s) used AND whether they are one- or two-sided<br>*Only common tests should be described solely by name; describe more complex techniques in the Methods section.*
☒	☐	A description of all covariates tested
☒	☐	A description of any assumptions or corrections, such as tests of normality and adjustment for multiple comparisons
☐	☒	A full description of the statistical parameters including central tendency (e.g. means) or other basic estimates (e.g. regression coefficient) AND variation (e.g. standard deviation) or associated estimates of uncertainty (e.g. confidence intervals)
☐	☒	For null hypothesis testing, the test statistic (e.g. *F*, *t*, *r*) with confidence intervals, effect sizes, degrees of freedom and *P* value noted<br>*Give P values as exact values whenever suitable.*
☒	☐	For Bayesian analysis, information on the choice of priors and Markov chain Monte Carlo settings
☒	☐	For hierarchical and complex designs, identification of the appropriate level for tests and full reporting of outcomes
☒	☐	Estimates of effect sizes (e.g. Cohen's *d*, Pearson's *r*), indicating how they were calculated

Our web collection on statistics for biologists contains articles on many of the points above.

Software and code

Policy information about availability of computer code

Data collection	IHC,IF and HE images were captured using Zeiss Zen Blue edition (version 3.4) .For flow cytometry analysis data were collected using a flow cytometer (CytoFLEX, Beckman Coulter) .Western Blot data were collected by Tanon 5200 intelligent imaging system.For real-time qPCR was performed using PerfectStart® Green qPCR SuperMix (AQ601， TransGen Biotech), by a Bio-Rad CXF96 real-time system (Bio-Rad, USA).
Data analysis	NovoExpress 2.0,Primer Premier Versions 5.00,Cell Ranger Versions 4.0.0,Seurat Versions 4.0.0,DoubletFinder Versions 2.0.3,SCENIC version 1.2.1,Limma Versions 3.52.2,Cellchat Versions 1.4.0,CellPhoneDB Versions 2.1.7.

For manuscripts utilizing custom algorithms or software that are central to the research but not yet described in published literature, software must be made available to editors and reviewers. We strongly encourage code deposition in a community repository (e.g. GitHub). See the Nature Portfolio guidelines for submitting code & software for further information.

Data

Policy information about availability of data

All manuscripts must include a data availability statement. This statement should provide the following information, where applicable:

- Accession codes, unique identifiers, or web links for publicly available datasets
- A description of any restrictions on data availability
- For clinical datasets or third party data, please ensure that the statement adheres to our policy

The raw sequencing data reported in this paper (8 mouse bladder cancer scRNA-seq data) has been deposited in the Genome Sequence Archive in the National

Genomics Data Center under the accession number CRA008674. （https://ngdc.cncb.ac.cn/） Due to data privacy laws, raw sequencing data can be used for non-commercial purposes under controlled access and access can be obtained through a request from the corresponding author. The request will be approved within 1 week and the users will then be provided with a download link valid for 1 year to download the raw data.

Research involving human participants, their data, or biological material

Policy information about studies with human participants or human data. See also policy information about sex, gender (identity/presentation), and sexual orientation and race, ethnicity and racism.

Reporting on sex and gender	N/A, Information not collected
Reporting on race, ethnicity, or other socially relevant groupings	N/A, Information not collected
Population characteristics	We collected 93 tumor tissue specimens from patients with BLCA who underwent surgery at the Fourth Medical Center of the PLA General Hospital.A commercial tissue microarray containing 63 BLCA and 16 para-tumor tissues from Shanghai OUTDO BIOTECH Co., Ltd.
Recruitment	We collected 93 tumor tissue specimens from patients with BLCA who underwent surgery at the Fourth Medical Center of the PLA General Hospital.A commercial tissue microarray containing 63 BLCA and 16 para-tumor tissues from Shanghai OUTDO BIOTECH Co., Ltd.
Ethics oversight	Tumor samples were collected with the patients' written informed consent and approved by the Human Research Ethics Committee of the Fourth Medical Center of PLA General Hospital. Human bladder cancer tissues arrays are purchased from Shanghai OUTDO Biotech Co., Ltd.

Note that full information on the approval of the study protocol must also be provided in the manuscript.

Field-specific reporting

Please select the one below that is the best fit for your research. If you are not sure, read the appropriate sections before making your selection.

☒ Life sciences ☐ Behavioural & social sciences ☐ Ecological, evolutionary & environmental sciences

For a reference copy of the document with all sections, see nature.com/documents/nr-reporting-summary-flat.pdf

Life sciences study design

All studies must disclose on these points even when the disclosure is negative.

Sample size	Groups of C57BL/6N mice experiments were performed using n ≥ 6 biologically independent samples. Where possible, sample sizes were selected according to established protocols in accepted standards within the scientific community
Data exclusions	No data was excluded in this study.
Replication	Consistency of results was verified by repeated biological independent experiments. Biologically independent experiments with consistent results and sample sizes are shown in the legend in Fig.
Randomization	The mice were randomly put into separate/groups cages for experiments.
Blinding	Although we were not able to implement blinding in our experiments, we conducted these experiments using fixed parameters within the same experiment (i.e., data from both the control and experimental groups were collected within the same parameters). With this in mind, we have largely avoided the influence of human eyes and subjectivity on the conclusions of this study.

Reporting for specific materials, systems and methods

We require information from authors about some types of materials, experimental systems and methods used in many studies. Here, indicate whether each material, system or method listed is relevant to your study. If you are not sure if a list item applies to your research, read the appropriate section before selecting a response.

Materials & experimental systems

n/a	Involved in the study	
☐	☒	Antibodies
☐	☒	Eukaryotic cell lines
☒	☐	Palaeontology and archaeology
☐	☒	Animals and other organisms
☒	☐	Clinical data
☒	☐	Dual use research of concern
☒	☐	Plants

Methods

n/a	Involved in the study	
☒	☐	ChIP-seq
☐	☒	Flow cytometry
☒	☐	MRI-based neuroimaging

Antibodies

Antibodies used

Treatment
InVivoMAb rat IgG1 isotype control BioXcell Cat#BE0088;
InVivoMAb anti-mouse CD276 (B7-H3) BioXcell Cat#BE0124;
InVivoMAb anti-mouse PD-1 (CD279) BioXcell Cat# BE0146;
InVivoPlus mouse IgG2a isotype BioXcell Cat#BP0085;
InVivoPlus anti-mouse CD8α BioXcell Cat#BP0061;
InVivoPlus anti-mouse CD4 BioXcell Cat#BP00031;
IHC, IF and WB
Rabbit polyclonal anti-MKI67 (Novus Cat# NB500-170) has been previously validated by the manufacturer (more information isavailable at https://www.novusbio.com/products/ki67-mki67-antibody_nb500-170) and cited at least 160 times.
Rabbit monoclonal anti-CD276 (CST Cat#14058) has been previously validated by the manufacturer (more information isavailable at https://www.cellsignal.com/products/primary-antibodies/b7-h3-d9m2l-xp-rabbit-mab/14058) and cited at least 40 times.
Rabbit polyclonal anti-CD276 (abclonal Cat#A17216) has been previously validated by the manufacturer (more information isavailable at https://abclonal.com.cn/catalog/A17216) .
Rabbit polyclonal anti-Cleaved Caspase-3 (CST Cat# 9661S0) has been previously validated by the manufacturer (more information isavailable at https://www.cellsignal.com/products/primary-antibodies/cleaved-caspase-3-asp175-antibody/9661) and cited at least 9591 times.
Rabbit polyclonal anti-MER1 (Proteintech Cat# 27044-1-AP) has been previously validated by the manufacturer (more information isavailable at https://www.ptglab.co.jp/Products/EMR1---F4-80-Antibody-27044-1-AP.htm) and cited at least 29 times.
Rabbit monoclonal anti-CD8α (abcam Cat# ab217344) has been previously validated by the manufacturer (more information isavailable at https://www.abcam.com/products/primary-antibodies/cd8-alpha-antibody-epr21769-ab217344.html) and cited at least 96 times.
Rabbit monoclonal anti-CD163 (abcam Cat# ab182422) has been previously validated by the manufacturer (more information isavailable at https://www.abcam.com/products/primary-antibodies/cd163-antibody-epr19518-ab182422.html) and cited at least 286 times.
Rabbit polyclonal anti-INOS (abcam Cat# ab283655) has been previously validated by the manufacturer (more information isavailable at https://www.abcam.com/products/primary-antibodies/inos-antibody-rm1017-ab283655.html) and cited at least 6 times.
Rabbit polyclonal anti-GZMA （abclonal Cat# A6231) has been previously validated by the manufacturer (more information isavailable at https://abclonal.com.cn/catalog/A6231) and cited at least 2 times.
Rabbit polyclonal anti-LAPTM5 (ImmunoWay Cat# YN4729) has been previously validated by the manufacturer (more information isavailable at http://www.immunoway.com/CHome/22/YN4729) .
Rabbit polyclonal anti-CLTC (Proteintech Cat# 26523-1-AP)has been previously validated by the manufacturer (more information isavailable at https://www.ptgcn.com/Products/CLTC-Antibody-26523-1-AP.htm) and cited at least 7 times.
Rabbit polyclonal anti-LIPA (Proteintech Cat# 12956-1-AP) has been previously validated by the manufacturer (more information isavailable at https://www.ptgcn.com/products/LIPA-Antibody-12956-1-AP.htm) and cited at least 7 times.
Rabbit polyclonal anti-LAMP2 (Proteintech Cat# 27823-1-AP) has been previously validated by the manufacturer (more information isavailable at https://www.ptgcn.com/products/LAMP2-Antibody-27823-1-AP.htm) and cited at least 40 times.
Rabbit polyclonal anti-CXCR4 (Proteintech Cat# 11073-2-AP) has been previously validated by the manufacturer (more information isavailable at https://www.ptgcn.com/Products/CXCR4-Antibody-11073-2-AP.htm) and cited at least 38 times.
Rabbit polyclonal anti-CXCL9 (Proteintech Cat# 22355-1-AP)has been previously validated by the manufacturer (more information isavailable at https://www.ptglab.co.jp/Products/MIG-Antibody-22355-1-AP.htm) and cited at least 5 times.
Rabbit polyclonal anti-CX3CR1 (ImmunoWay Cat# YT5112) has been previously validated by the manufacturer (more information isavailable at http://www.immunoway.com.cn/CHome/22/YT5112) .
Rabbit polyclonal anti-CCR1 (Thermo Fisher Cat# PA1-41062) has been previously validated by the manufacturer (more information isavailable at https://www.thermofisher.cn/cn/zh/antibody/product/CCR1-Antibody-Polyclonal/PA1-41062) and cited at least 3 times.
Rabbit polyclonal anti-CCR5 (ImmunoWay Cat# YT6108) has been previously validated by the manufacturer (more information isavailable at http://www.immunoway.com.cn/CHome/22/YT6108).
Rabbit polyclonal anti-AXL (Affinity Cat# AF7793) has been previously validated by the manufacturer (more information isavailable at https://affbiotech.cn/goods-14439-AF7793-AXL_Antibody.html) and cited at least 1 times.
Rabbit polyclonal anti-JUN (Proteintech Cat# 24909-1-AP) has been previously validated by the manufacturer (more information isavailable at https://www.ptgcn.com/Products/JUN-Antibody-24909-1-AP.htm) and cited at least 50 times.
Rabbit monoclonal anti-MERTK (abcam Cat# ab300136) has been previously validated by the manufacturer (more information isavailable at https://www.abcam.cn/products/primary-antibodies/mertk-antibody-epr26359-12-ab300136.html).
Rabbit monoclonal anti-GAPDH (CST Cat#2118S) has been previously validated by the manufacturer (more information isavailable at https://www.cellsignal.cn/products/primary-antibodies/gapdh-14c10-rabbit-mab/2118) and cited at least 7799 times.
anti-rabbit horseradish peroxidase-conjugated secondary antibody (Proteintech Cat# SA00001-2) has been previously validated by

the manufacturer (more information isavailable at https://www.ptglab.co.jp/products/HRP-conjugated-Affinipure-Goat-Anti-Rabbit-IgG-H-L-secondary-antibody.htm) and cited at least 7708 times.
Flow Cytometry
Anti-Mouse CD11C PE-CY7 (Thermo Fisher Cat#25-0114-81) has been previously validated by the manufacturer (more information isavailable at https://www.thermofisher.cn/cn/zh/antibody/product/CD11c-Antibody-clone-N418-Monoclonal/25-0114-82) and cited at least 175 times.
Anti-Mouse F4/80 PCP-CY5.5 (Thermo Fisher Cat#45-4801-80) has been previously validated by the manufacturer (more information isavailable at https://www.thermofisher.cn/cn/zh/antibody/product/F4-80-Antibody-clone-BM8-Monoclonal/45-4801-82) and cited at least 198 times.
Anti-Mouse Ly-6G (Gr-1) Alexa Fluor® 700 (Thermo Fisher Cat#56-5931-82) has been previously validated by the manufacturer (more information isavailable at https://www.thermofisher.cn/cn/zh/antibody/product/Ly-6G-Ly-6C-Antibody-clone-RB6-8C5-Monoclonal/56-5931-82) and cited at least 110 times.
Annexin V-APC/7-AAD Apoptosis kit LIANKE Cat#AP105;
Anti-Mouse MHC Class II (I-A/I-E) eFluor® 450 (Thermo Fisher Cat#48-5321-82)has been previously validated by the manufacturer (more information isavailable at https://www.thermofisher.cn/cn/zh/antibody/product/MHC-Class-II-I-A-I-E-Antibody-clone-M5-114-15-2-Monoclonal/48-5321-82) and cited at least 162 times.
Anti-Mouse CD45 PE-CY7 (Tonbo Cat#60-0451)has been previously validated by the manufacturer (more information isavailable at https://cytekbio.com/products/pe-cyanine7-anti-mouse-cd45-30-f11?variant=40581201362980) .
Anti-Mouse CD3 APC (Tonbo Cat#20-0032) has been previously validated by the manufacturer (more information isavailable at https://www.netascientific.com/primary-antibodies/tb-20-0032-u100) .
Anti-Mouse CD4 violet FluorTM 450 (Tonbo Cat#75-0041) has been previously validated by the manufacturer (more information isavailable at https://www.biocompare.com/9776-Antibodies/9246604-violetFluor-8482-450-Anti-Mouse-CD4-GK1-5/) and cited at least 4 times.
Anti-Mouse CD8a FITC (Tonbo Cat#35-0081) has been previously validated by the manufacturer (more information isavailable at https://www.thermofisher.cn/cn/zh/antibody/product/CD8a-Antibody-clone-53-6-7-Monoclonal/35-0081-82) and cited at least 82 times.
Ghost DyeTM Red 780 (Tonbo Cat#13-0865) has been previously validated by the manufacturer (more information isavailable at https://cytekbio.com/products/ghost-dye-red-780) and cited at least 22 times.

Validation

All primary antibodies were used according to the manufacturer's recommended dilutions and buffers. All antibodies purchased were verified by the company from whom they were obtained according to the manufacturer's website.

Eukaryotic cell lines

Policy information about cell lines and Sex and Gender in Research

Cell line source(s)

MB49(Mouse bladder cancer cell line);MBT2 （Mouse Bladder Tumor line-2)

Authentication

MBT2 cells were obtained from the First Affiliated Hospital of Anhui Medical University, while MB49 cells were purchased from EMD Millipore (Merck, Cat# SCC148).

Mycoplasma contamination

All the cells were tested negative for mycoplasma.

Commonly misidentified lines
(See ICLAC register)

No commonly misidentified cell lines were used.

Animals and other research organisms

Policy information about studies involving animals; ARRIVE guidelines recommended for reporting animal research, and Sex and Gender in Research

Laboratory animals

WT C57BL/6, Cd276-/-, Cd276fl/fl and Rosa-Cd276 mice were purchased from The GemPharmatech (Nanjing, China). Rosa-tdTomato (007914) mice were purchased from The Jackson Laboratory (Bar Harbor, ME, USA). Axl-/- mouse strain was a gift from Dr. Guoqiang Yuan (The Second Hospital of Lanzhou University). Lyz2-Cre (B6.129P2-Lyz2tml (cre) lfo/J) mouse were kindly given by Dr. Xiaojun Xia (Sun Yat-sen university Cancer Center). All animals in this study were maintained under specific pathogen free conditions and housed under a 12-hour light/dark cycle and given ad libitum access to food and water. All animal experiments were approved by the Animal Ethics Committee of the First Affiliated Hospital of Sun Yet-sen University (protocol number SYSU-IACUC-2021-000138). For bladder cancer induction, male mice at least 6 weeks old received BBN at a dose of 0.05% in drinking water as previously describe

Wild animals

No wild animals were used in this study.

Reporting on sex

The mice used in the study were all male, because the incidence of bladder cancer is much higher in men than in women (PMID: 27370177).

Field-collected samples

The study did not invole samples collected from field.

Ethics oversight

All animal experiments were approved by the Animal Ethics Committee of the First Affiliated Hospital of Sun Yet-sen University (protocol number SYSU-IACUC-2020-000336).

Note that full information on the approval of the study protocol must also be provided in the manuscript.

Flow Cytometry

Plots

Confirm that:

☒ The axis labels state the marker and fluorochrome used (e.g. CD4-FITC).

☐ The axis scales are clearly visible. Include numbers along axes only for bottom left plot of group (a 'group' is an analysis of identical markers).

☐ All plots are contour plots with outliers or pseudocolor plots.

☒ A numerical value for number of cells or percentage (with statistics) is provided.

Methodology

Sample preparation	As stated above, single cell suspensions of mouse marrow, spleen, and xenograft were created. Before staining, cells were resuspended at a concentration of 1 × 107 cells/mL after being rinsed in staining buffer (2% bovine growth serum in PBS). Cells were stained with antibodies and then resuspended in staining buffer for 2 h at 4°C to achieve extracellular staining. Anti-Mouse CD11C PE-CY7 (25-0114-81, Thermo Fisher); Anti-Mouse F4/80 PCP-CY5.5 (45-4801-80, Thermo Fisher); Anti-Mouse Ly-6G (Gr-1) Alexa Fluor® 700 (56-5931-82, Thermo Fisher); Annexin V-APC/7-AAD Apoptosis kit (AP105, LIANKE); Anti-Mouse MHC Class II (I-A/I-E) eFluor® 450 (48-5321-82, Thermo Fisher); Anti-Mouse CD45 PE-CY7(60-0451, Tonbo); Anti-Mouse CD3 APC (20-0032, Tonbo); Anti-Mouse CD4 violet FluorTM 450 (75-0041, Tonbo); Anti-Mouse CD8a FITC(35-0081, Tonbo) and Ghost DyeTM Red 780 (13-0865, Tonbo) were the antibodies and dyes used for flow cytometry (CytoFLEX, Beckman Coulter). Data were examined using NovoExpress software, and samples were examined using a flow cytometer (CytoFLEX, Beckman Coulter) (version 2.0).
Instrument	FC Cell Sorters: BD LSRFOrtessa.<br>FC Analysers: CytoFLEX, Beckman Coulter
Software	NovoExpress 2.0 and CytoFLEX2.0.
Cell population abundance	According to the estimated size and particle size of the forward and lateral scattering cells, as well as the accompanying positive or negative signals, the cell population is established for flow analysis.
Gating strategy	Gating strategies were illustrated in supplementary figures.

☒ Tick this box to confirm that a figure exemplifying the gating strategy is provided in the Supplementary Information.
