# Supplementary material for: CD276-dependent efferocytosis by tumor-associated macrophages promotes immune evasion in bladder cancer
Source: Nat Commun. 2024 Apr 1;15:2818. doi: 10.1038/s41467-024-46735-5 (PMC10985117; doi:10.1038/s41467-024-46735-5)
Supplement: Supplementary file 11 — Source Data [file 41467_2024_46735_MOESM11_ESM.zip › WB.pptx]

## Slide 1
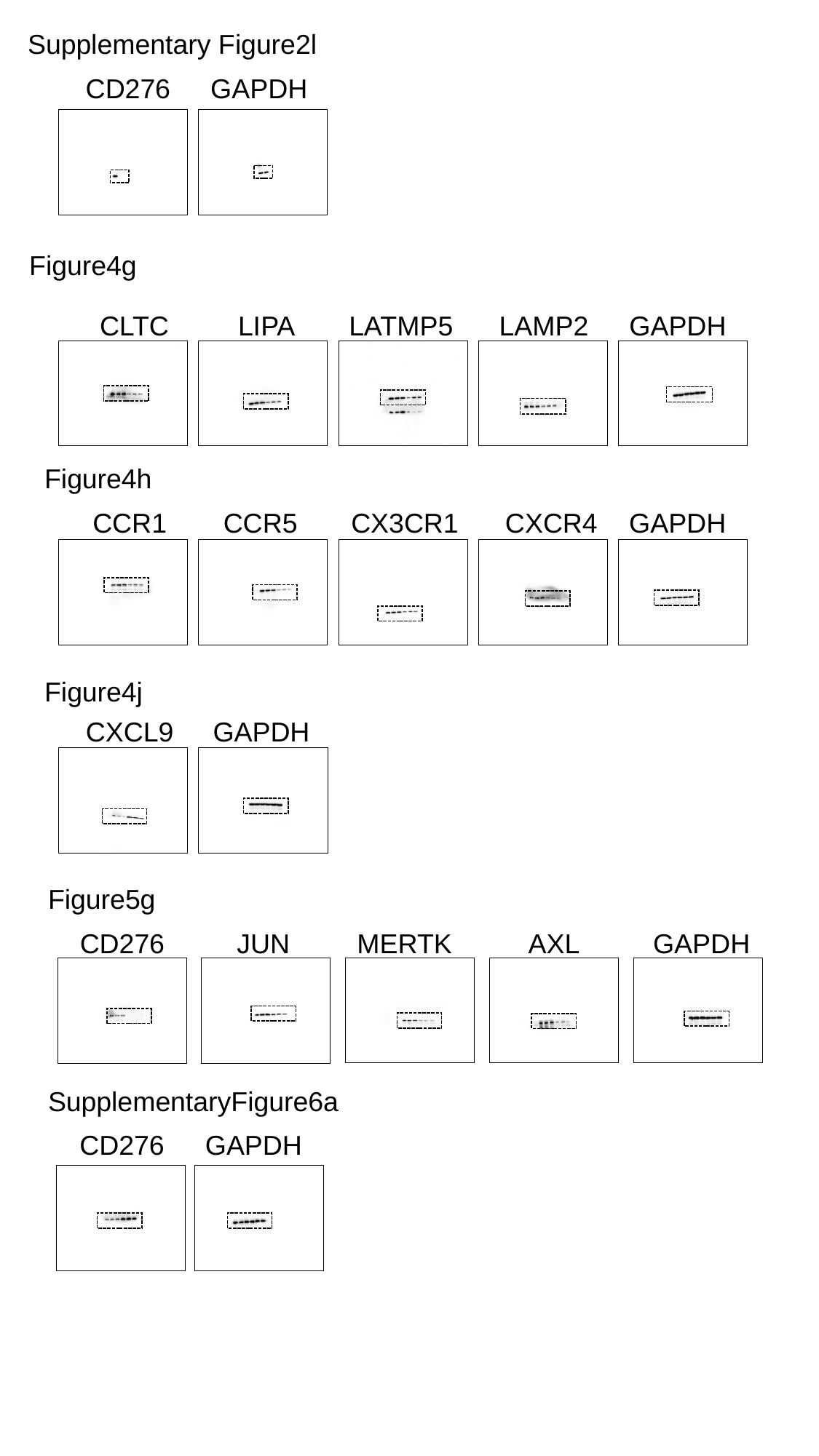

Supplementary Figure2l
CD276
GAPDH
Figure4g
CLTC
LIPA
LATMP5
LAMP2
GAPDH
Figure4h
CCR1
CCR5
CX3CR1
CXCR4
GAPDH
Figure4j
CXCL9
GAPDH
Figure5g
JUN
AXL
CD276
MERTK
GAPDH
SupplementaryFigure6a
CD276
GAPDH
